# The Reliability and Validity of an Isometric Knee Strength Measurement Device in Older Adult Individuals

**DOI:** 10.3390/s25102981

**Published:** 2025-05-08

**Authors:** Jae-Soo Hong, Jeong-Bae Ko, Myeong-Min Ju, Byoung-Kwon Lee, Dae-Sung Park, Su-Ha Lee

**Affiliations:** 1 Regional Industrial Innovation Department, Korea Institute of Industrial Technology, Cheonan-si 31056, Republic of Korea; jshong94@kitech.re.kr (J.-S.H.); duckbaeya@kitech.re.kr (J.-B.K.); 2 Department of Physical Therapy, Konyang University, Daejeon 35365, Republic of Korea; juaudals6774@naver.com (M.-M.J.); lbk6326@konyang.ac.kr (B.-K.L.); daeric@konyang.ac.kr (D.-S.P.); 3 Department of Physical Therapy, Sahmyook University, Seoul 01795, Republic of Korea

**Keywords:** knee strength assessment, dynamometer, sarcopenia, isometric strength

## Abstract

**Highlights:**

**What are the main findings?**

- The IB-LS device demonstrated excellent test–retest reliability (ICC = 0.856–0.987) in measuring isometric knee strength in older adults.
- Validity analysis showed strong agreement between IB-LS and both isometric and isokinetic strength values from the CSMI device (ICC > 0.77).

**What is the implication of the main finding?**

- The IB-LS provides a reliable and portable alternative to conventional isokinetic dynamometers for knee strength assessment.
- This device enables simple and efficient strength evaluations in clinical and community settings for older populations.

**Abstract:**

This study aims to evaluate the reliability and validity of the Leg Strength Analyzer (IB-LS) in assessing isometric knee flexion and extension strength in elderly adults, compare its performance with that of the CSMI dynamometer, and examine its agreement with isokinetic knee strength measurements. A total of 21 elderly participants (mean age: 65.10 ± 4.56 years) were recruited. Participants underwent knee flexion and extension strength assessments using both the IB-LS and CSMI devices, with isokinetic strength at 60°/s measured in a follow-up session at least one day later. The IB-LS demonstrated high test–retest reliability in elderly adults (ICC = 0.856–0.987). The validity analysis comparing IB-LS isometric peak torque with CSMI isometric peak torque showed moderate to high validity (ICC = 0.826–0.946). Furthermore, IB-LS isometric peak torque and CSMI isokinetic 60°/s peak torque demonstrated high agreement (ICC = 0.775–0.881), demonstrating its strong association with isokinetic strength assessments. Bland–Altman analysis revealed that mean differences between IB-LS and CSMI isometric peak torque values ranged from 13.2 to 93.5 Nm, with limits of agreement (LoA) spanning from −55.8 to 192.5 Nm. When comparing IB-LS isometric peak torque with CSMI isokinetic 60°/s peak torque, mean differences ranged from 31.0 to 56.0 Nm, with LoA from −28.5 to 138.9 Nm. The IB-LS is a reliable and valid tool for evaluating isometric knee strength in elderly adults. Its strong agreement with the CSMI dynamometer and close correlation with isokinetic strength measurements indicate that IB-LS can be a feasible alternative for assessing knee strength in clinical and research settings focused on elderly populations.

## 1. Introduction

The knee joint performs essential functions in daily activities such as walking, climbing stairs, and sitting, while lower limb muscle strength plays a crucial role in physical mobility and balance maintenance [1]. However, age-related declines in muscle mass and strength impair knee joint function, making independent daily living more challenging and increasing the risk of falls and physical disabilities [2]. Studies have reported that muscle strength decreases by approximately 15% per decade after the age of 50 and by more than 30% after the age of 70 [3]. The World Health Organization (WHO) estimates that the global population aged 60 and older will reach approximately 2.2 billion by 2050, highlighting the urgent need to manage health issues related to sarcopenia from a public health perspective [4]. This decline in muscle strength is not merely a natural consequence of aging but is considered part of the pathological process of sarcopenia, which is closely associated with physical function deterioration and increased mortality [5].

Sarcopenia is characterized by both quantitative and qualitative declines in skeletal muscle, leading to physical function deterioration, reduced gait speed, and impaired balance, ultimately making it difficult to maintain independent living [6]. In recognition of its clinical significance, the U.S. Centers for Disease Control and Prevention (CDC) and the World Health Organization (WHO) have designated sarcopenia as a disease with code M62.84, while South Korea officially classified it as a disease (M62.5) in the 2021 revision of the Korean Standard Classification of Diseases (KCD) [7]. Studies have reported that individuals with sarcopenia have a fourfold increased risk of developing physical disabilities in daily life, along with a two- to threefold higher risk of balance and gait impairments [8]. Consequently, the necessity of objective lower limb strength assessments for the early diagnosis and prevention of sarcopenia has been internationally emphasized, underscoring the need for the development of effective evaluation methods [9].

Isometric and isokinetic strength assessments are the two primary methods for evaluating lower limb muscle strength, each serving distinct purposes in assessing physical function [10]. An isometric strength measurement evaluates maximal muscle strength without changes in muscle length, making it useful for assessing neuromuscular control and muscle function [11]. In contrast, isokinetic strength measurement evaluates muscle strength at a constant angular velocity, allowing for the analysis of the force–velocity relationship of muscles, which is widely utilized in sports injury assessments and rehabilitation [12]. Previous studies have reported a high correlation between isometric and isokinetic strength assessments, with findings suggesting that isometric strength testing alone can provide an objective evaluation of physical function [13].

One of the most widely used devices for lower limb strength assessment is the CSMI (CSMI Humac Norm., Computer Sports Medicine Inc., Stoughton, MA, USA), which is recognized as the gold standard for isokinetic strength evaluation [14]. However, the high cost, complex measurement procedures, and substantial spatial requirements of this device often limit its applicability in clinical and research settings [15]. As a more efficient and cost-effective alternative, the Leg Strength Analyzer (IB-LS, InBody Inc., Seoul, Republic of Korea), recently developed by InBody, has gained attention. This device utilizes a load cell-based force measurement system designed to assess isometric strength during knee extension and flexion. However, systematic validation is required to determine whether the reliability and validity of the IB-LS are equivalent to those of the existing gold standard device, CSMI.

Although previous studies have reported a high correlation between isometric and isokinetic strength measurements [16], the degree of correlation between the isometric strength values obtained from the IB-LS and the isometric and isokinetic strength values measured by the CSMI has not been sufficiently validated. In particular, there is a lack of research on the reliability of the IB-LS in comparison with conventional isometric reference devices and whether its isometric strength measurements can be associated with isokinetic strength evaluation.

Therefore, this study aims to evaluate the test–retest reliability of isometric strength measurements obtained using the InBody IB-LS in older adults, verify its validity by comparing its isometric strength values with those measured by the CSMI device, and analyze the relationship between the isometric strength values measured by the IB-LS and the isokinetic strength values (60°/s) obtained from the CSMI device. Through this study, we aim to determine whether the IB-LS device is a reliable tool for lower limb strength assessment and to evaluate its clinical applicability by comparing it with conventional isokinetic strength assessment devices.

To achieve this, lower limb strength will be assessed using both the IB-LS and CSMI devices, with two repeated measurements conducted on the IB-LS to evaluate its reliability. Test–retest reliability will be assessed using Intraclass Correlation Coefficient (ICC) analysis. The validity of isometric strength measurements between the IB-LS and CSMI devices will be verified through paired *t*-tests and ICC analysis. Additionally, to examine the relationship between isometric strength values obtained from the IB-LS and isokinetic strength values (60°/s) measured by the CSMI device, paired *t*-tests and ICC analysis will be conducted. To quantitatively compare the differences between the two devices, Bland–Altman analysis will be utilized. Through this analysis, the mean difference, 95% confidence interval (CI), and limits of agreement (LoA) will be calculated to evaluate the level of agreement between the measurements obtained from both devices.

This study aims to determine whether the IB-LS device can serve as a reliable tool for assessing lower limb strength in older adults and to explore its applicability in clinical and research settings by analyzing its relationship with isokinetic strength measurements. Through this, we seek to evaluate the utility of the IB-LS as a practical alternative to conventional isokinetic strength assessment devices and propose a more convenient and effective approach for assessing lower limb strength in older adults.

## 2. Materials and Methods

### 2.1. General Characteristics

This study was conducted with 21 community-dwelling older adults residing in D Metropolitan City, South Korea. Participants were voluntarily recruited through local senior welfare centers and community organizations. Only individuals who fully understood the study’s purpose and procedures and provided written informed consent were included in the study.

The study participants were adults aged 60 to 79 years who had no history of orthopedic or neurological injuries in the past six months and were able to perform daily activities independently. Conversely, individuals who had undergone lower limb surgery within the past six months, had musculoskeletal or neurological conditions that made lower limb strength assessment difficult, or withdrew from the study due to dizziness or discomfort during the experiment were excluded. Although nutritional status, chronic diseases (such as diabetes or cardiovascular disease), and medication use were not part of the inclusion or exclusion criteria, we acknowledge that these factors may influence lower limb muscle strength in older adults. Their potential impact is addressed in the limitations section.

All study participants provided written informed consent after fully understanding the study’s purpose and procedures and were allowed to voluntarily withdraw at any time during the study. Additionally, the researchers closely monitored any potential discomfort or adverse effects that might occur during the study. This study was conducted with the approval of the Institutional Review Board (IRB) of K University (KYU-2023-11-046-003).

### 2.2. Procedures

This study was designed as a reliability and validity study to evaluate the reliability and validity of the InBody Leg Strength Analyzer (IB-LS, InBody Inc., Republic of Korea). The participants’ sex, age, height, weight, dominant leg, and lower limb length were recorded. Muscle mass was measured using a body composition analyzer (InBody 570, InBody Inc., Republic of Korea). The dominant leg was determined using a self-reported method based on the foot the participant primarily used. Lower limb length was measured as the distance from the anterior superior iliac spine (ASIS) to the medial malleolus.

Before knee strength assessment, participants performed a five-minute warm-up on an ergometer bicycle (Wattbike Pro, Wattbike Ltd., West Bridgford, UK) at an intensity of 50 watts. After completing the warm-up, they rested for five minutes before undergoing strength evaluation. Knee extension and flexion strength were assessed using the InBody Leg Strength Analyzer (IB-LS) and the CSMI dynamometer (Humac Norm Isokinetic Testing and Reha-bilitation System, Computer Sports Medicine Inc., USA). The order of isometric strength measurements using the two devices was randomly assigned using a computer-generated random number generator.

After completing the isometric strength assessments using the IB-LS and CSMI devices, an additional isokinetic strength evaluation was conducted using the CSMI device with at least a one-day interval to analyze the agreement between the isometric strength values measured by the IB-LS and the standardized isokinetic strength measurements. During the evaluation, participants performed knee extension and flexion strength assessments at a velocity of 60°/s, with each session consisting of five repetitions, and the peak value was recorded. Subsequently, the agreement between the isometric strength values measured by the IB-LS and the isokinetic strength values obtained from the CSMI device was analyzed to determine whether the IB-LS isometric strength assessment can serve as an equivalent alternative to isokinetic strength measurement.

All measurements in this study were consistently conducted by a single experienced physical therapist using standardized methods to ensure accuracy across assessments.

### 2.3. Knee Flexion and Extension Strength Assessment Device

**Figure 1:**
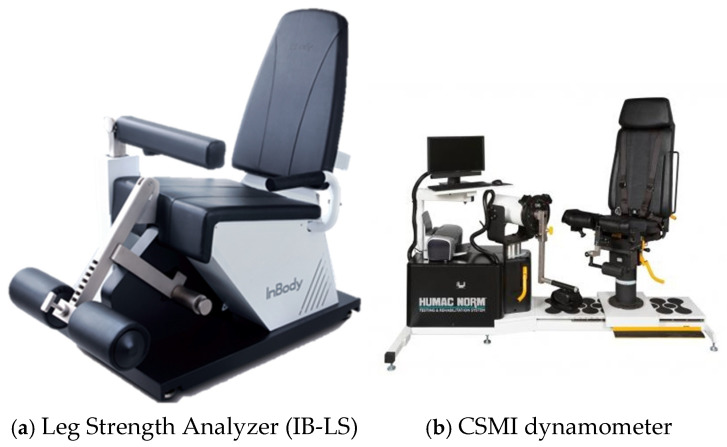
The devices of this experimental study: (**a**) Leg Strength Analyzer; (**b**) CSMI dynamometer.

The Leg Strength Analyzer is a device designed to assess lower limb strength and consists of a mechanical unit and a control unit. The mechanical unit includes a seat, leg fixation components, a leg strength measurement module, and handles, with an adjustable backrest for positioning. The leg strength measurement module has a total length of 470 mm and can be adjusted in nine stages according to leg length. When the participant is seated, the measurement module is positioned at the center of both legs, and a load cell is attached to measure the force exerted during knee flexion and extension. The control unit consists of a power system and a 7-inch touchscreen display, providing real-time measurement values. The dimensions of the IB-LS mechanical unit are 610 × 1370 × 1065 mm, with a total weight of 108 kg. The control unit measures 440 × 440 × 990 mm and weighs 8.5 kg. The load cell has a measurement range of 5 to 150 kg, with precision up to one decimal place. The recorded measurement values include peak force and peak torque (Figure 1).

The CSMI dynamometer is a device designed for isokinetic and isometric strength assessments and is widely recognized as the standard equipment for lower limb strength measurement. The mechanical unit consists of a dedicated seat, leg fixation components, handles, stabilization straps, and a resistance module. During the assessment, the participant sits on the seat, grips the handles with both hands, and secures their torso and pelvis using adjustable stabilization belts. The device measures muscle strength by providing consistent resistance at a preset angular velocity using a motor-based resistance system. This mechanism ensures that participants exert maximum force at the designated angular velocity, allowing for precise evaluation of both isokinetic and isometric strength.

The control unit includes a touchscreen display and a power system, providing real-time measurement data. The movement speed can be adjusted within a range of 1–500°/s, and in this study, the isokinetic strength assessment was conducted at a velocity of 60°/s. The dimensions and weight of the CSMI dynamometer vary by model, and the model used in this study had a mechanical unit size of 910 × 1270 × 1220 mm and a weight of 145 kg. The recorded measurement values include peak force and peak torque (Figure 1).

### 2.4. Statistical Analysis

To compare the general characteristics of the study participants, a Chi-square test (χ^2^ test) and an independent *t*-test were performed. To assess the test–retest reliability of the IB-LS device, each participant’s knee flexion and extension strength were measured three times, and the peak force (kg) values were obtained. Intraclass Correlation Coefficient (ICC) analysis was then conducted to evaluate reliability. The reliability levels were classified according to the criteria established by Rankin et al. [17], where an ICC value of ≤0.25 was considered very low, 0.26–0.49 as low, 0.50–0.69 as moderate, 0.70–0.89 as high, and 0.90–1.00 as very high.

To evaluate the validity of isometric strength measurements between the IB-LS and CSMI devices, a paired *t*-test and ICC analysis were conducted. In this process, the force (kg) values measured by the IB-LS were converted into peak torque (Nm) values by multiplying them by the participant’s lower leg length (m), defined as the distance from the center of the knee to the resistance application site. The converted peak torque values were then compared with the isometric strength measurements obtained from the CSMI device. Additionally, a paired *t*-test and ICC analysis were performed to assess the validity of the isometric peak torque values measured by the IB-LS in comparison with the isokinetic (60°/s) peak torque values obtained from the CSMI device.

To assess the agreement between the measurements obtained from the two devices, a Bland–Altman analysis was conducted. The mean difference, 95% confidence interval (CI), and limits of agreement (LoA) were calculated to examine the consistency of the measurements. According to the criteria established by Bland & Altman [18], a mean difference closer to zero and a narrower LoA range indicate a higher level of agreement between the two devices, suggesting minimal measurement bias and high reproducibility. The mean difference and LoA range between IB-LS and CSMI measurements were numerically compared, and the Bland–Altman analysis results for isometric and isokinetic strength assessments were analyzed separately to evaluate the level of agreement between the two devices.

Statistical analyses were performed using SPSS 22.0 (SPSS Inc., Chicago, IL, USA) and MedCalc version 20.1 (MedCalc Software Ltd., Ostend, Belgium). The significance level for all analyses was set at 0.05.

## 3. Results

A total of 21 older adults (11 males and 10 females) participated in this study, with a mean age of 65.10 ± 4.56 years. The isokinetic strength assessment was conducted at least one day after the initial evaluation, with 17 participants (7 males and 10 females) completing the second measurement. Four participants who did not attend the follow-up assessment were excluded from the analysis (Table 1). No adverse reactions were observed in any participants during the experimental procedures.

The test–retest reliability assessment of the IB-LS device demonstrated high reliability across all parameters (ICC = 0.856–0.987) (Table 2). The ICC value for left knee flexion was 0.982, while the ICC value for right knee extension was 0.856. A comparison of the isometric strength measurements between the IB-LS and CSMI devices showed an ICC range of 0.826–0.946 across all parameters (Table 3). The ICC value for left knee flexion was 0.946, while the ICC value for left knee extension was 0.826. The ICC values for right knee flexion and extension were 0.946 and 0.883, respectively. The mean differences in isometric strength values between the two devices were statistically significant (all *p* < 0.05), with a mean difference of 12.04 Nm for left knee flexion and 7.38 Nm for right knee flexion. A comparison between the isometric strength values measured by the IB-LS and the isokinetic (60°/s) strength values obtained from the CSMI device showed an ICC range of 0.775–0.881 (Table 4). The ICC value for left knee flexion was 0.881, while the ICC value for left knee extension was 0.847. The ICC values for right knee flexion and extension were 0.807 and 0.775, respectively. The mean difference between IB-LS and CSMI isokinetic (60°/s) strength measurements was 16.39 Nm for left knee flexion and 43.9 Nm for right knee extension.

**Figure 2:**
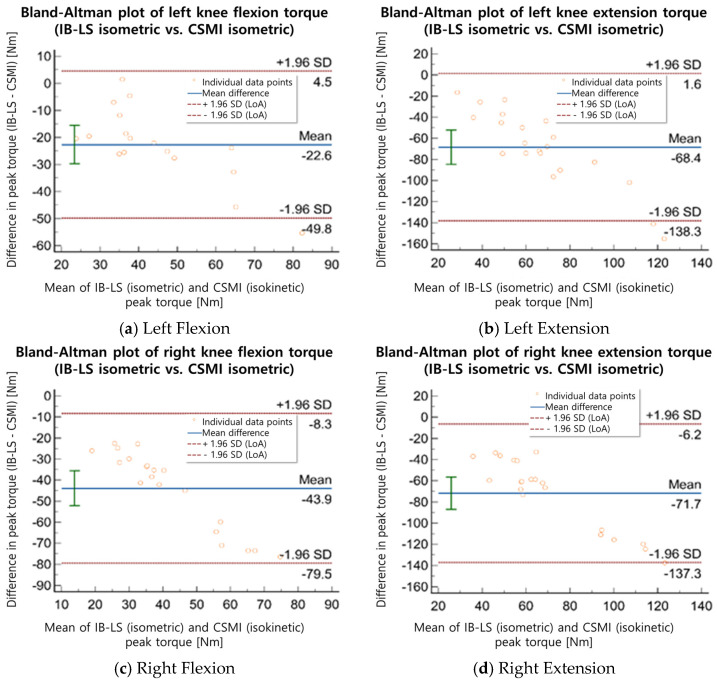
A graph comparing the mean difference in knee flexion/extension isometric peak torque between IB-LS and CSMI.

**Figure 3:**
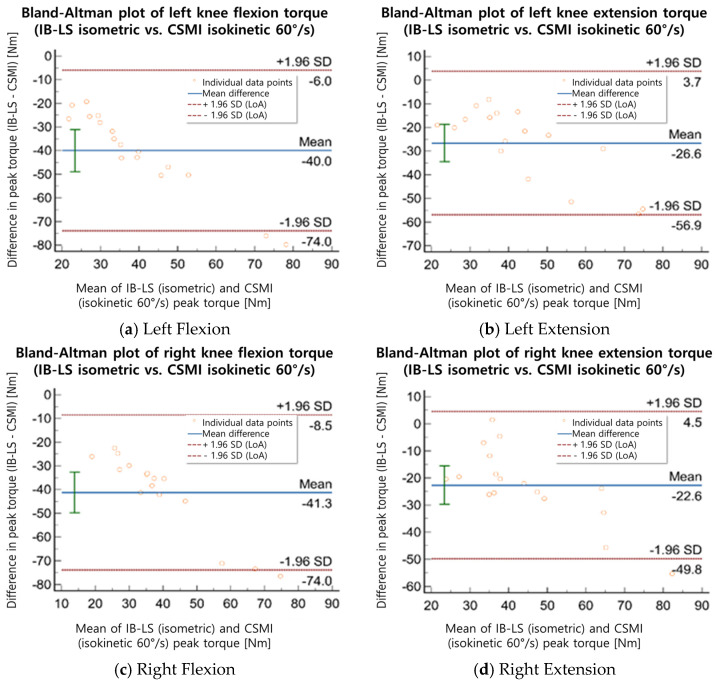
A graph comparing the mean difference between the elderly group knee flexion/extension isometric/isometric 60°/s peak torque between IB-LS and CSMI.

The Bland–Altman analysis revealed that the mean difference in isometric strength measurements between the IB-LS and CSMI devices ranged from 13.2 to 93.5 Nm (Table 5, Figure 2 and Figure 3). The mean difference between the IB-LS isometric peak torque values and the CSMI isokinetic (60°/s) peak torque values was 31.0–56.0 Nm. For left knee flexion (isometric), the mean difference was 13.2 Nm (LoA: −55.8 to 82.2 Nm), while for left knee extension (isometric), the mean difference was 74.7 Nm (LoA: −6.2 to 155.6 Nm). In isokinetic assessments, the mean difference for left knee flexion was 38.2 Nm (LoA: −16.24 to 92.7 Nm), and for left knee extension, it was 48.4 Nm (LoA: −11.71 to 108.4 Nm). For right knee flexion (isometric), the mean difference was 28.1 Nm (LoA: −34.5 to 90.8 Nm), while for right knee extension (isometric), the mean difference was 93.5 Nm (LoA: −5.6 to 192.5 Nm). In isokinetic assessments, the mean difference for right knee flexion was 31.0 Nm (LoA: −28.46 to 90.6 Nm), and for right knee extension, it was 56.0 Nm (LoA: −26.91 to 138.9 Nm). The IB-LS device demonstrated high test–retest reliability across all parameters (ICC = 0.856–0.987), while the isometric strength measurements compared with the CSMI device showed an ICC range of 0.826–0.946. The ICC values between IB-LS and CSMI isokinetic (60°/s) measurements ranged from 0.775 to 0.881. The Bland–Altman analysis indicated that the mean difference in strength measurements between the two devices ranged from 13.2 to 93.5 Nm.

## 4. Discussion

This study analyzed the reliability and validity of the IB-LS device for assessing isometric and isokinetic strength in knee flexion and extension. Previous studies have identified knee strength as a key indicator of lower limb function, and the relationship between isometric and isokinetic strength provides essential insights for clinical assessments and rehabilitation program design. Therefore, this study aimed to verify whether the isometric strength assessment using the IB-LS demonstrates reliability and validity comparable to the conventional CSMI isokinetic device.

The test–retest reliability of the IB-LS device was confirmed with an ICC range of 0.856–0.987, indicating that the device provides consistent results when repeated measurements are conducted on the same participants. Additionally, high ICC values were observed for both knee flexion and extension, supporting the use of IB-LS as a reliable assessment tool. This finding aligns with previous studies, which emphasizes that knee strength measurements must maintain reliability across repeated assessments and that isometric strength measurements should exhibit minimal variation between trials. Isometric strength assessment is generally known to have lower measurement error and higher reproducibility compared to dynamic strength assessments, and the results of this study further support this notion.

The peak strength of knee extension is measured at approximately 60°, while the peak strength of knee flexion is highest around 30°. Therefore, isokinetic strength assessment at 60°/s is commonly used to evaluate muscle strength, whereas isokinetic assessment at 180°/s is typically utilized to assess muscular endurance. In this study, the measurement posture of the participants was standardized such that full knee extension was defined as 0°, with knee flexion measured at 30° and knee extension at 60° [19]. During the measurement, a positional deviation of up to 10° in knee flexion and extension was observed, potentially resulting from individual anatomical differences or variations in participant posture during stabilization. Assessing muscle strength is essential for minimizing potential risks and evaluating an individual’s ability to return to daily activities after an injury [20,21,22]. This is partiularly important for determining the effectiveness of rehabilitation programs and quantitatively monitoring a patient’s recovery progress [21]. Therefore, accurate strength measurement plays a crucial role in supporting functional recovery and establishing personalized rehabilitation plans.

The validity of isometric strength measurements between the IB-LS and CSMI devices was confirmed with an ICC range of 0.799–0.946, indicating a high level of agreement between the two devices. This suggests that the isometric strength values measured by the IB-LS closely align with those obtained from the standardized strength assessment device, CSMI, supporting its reliability as an assessment tool in both clinical and research settings. The correlation between isometric strength values measured by the IB-LS and isokinetic (60°/s) strength values obtained from the CSMI device was found to be ICC = 0.775–0.869. However, since only isokinetic strength at 60°/s was assessed in this study, the comparison may not fully represent the relationship across different contraction speeds. Previous studies have shown that measurements at 180°/s are commonly used to assess muscular endurance. Future research is warranted to investigate whether similar relationships hold at higher isokinetic velocities. This result indicates that isometric strength has a substantial relationship with isokinetic strength and may have potential applications in supplementing isokinetic assessments or predicting muscle strength. Isometric strength measurement offers practical advantages, as it can be conducted with a simpler measurement process and lower cost compared to isokinetic assessments. Therefore, it may serve as a viable alternative in environments where isokinetic strength assessment is challenging to implement.

The Bland–Altman analysis showed that the mean difference in isometric strength measurements between the IB-LS and CSMI devices was 13.2 ± 3.5 Nm, with a LoA range of −55.8 to 192.5 Nm. Although the mean differences suggest acceptable agreement between the two measurement modalities, the relatively wide limits of agreement (e.g., −5.6 to 192.5 Nm for right knee extension) indicate substantial individual variability. Such discrepancies may have practical implications in clinical settings, particularly for older adults with compromised neuromuscular function or post-operative conditions. Accordingly, the interpretation of these results should be approached with clinical caution. These wide limits of agreement, particularly in right knee extension, suggest that while the average agreement is acceptable, substantial variation may occur at the individual level. Such variation could be clinically significant, especially in fragile or post-surgical older adults, and should be considered when interpreting the data in applied settings.

Additional evaluation may be necessary to determine whether any measurement bias is present. These discrepancies may be attributed to several factors, including structural differences in fixation methods between the two devices (e.g., strap-based vs. rigid), variability in participants’ effort during measurement, and slight misalignments in joint angle positioning. Such factors can influence torque output and should be considered when interpreting between-device differences. The mean difference between the IB-LS isometric peak torque values and the CSMI isokinetic (60°/s) peak torque values was 31.0 ± 6.0 Nm, with a LoA range of −28.5 to 138.9 Nm. This suggests that isometric strength may not only serve as a measure of static strength but also have a significant relationship with isokinetic strength.

Previous studies on patients who underwent total knee arthroplasty reported that isometric torque was 128 ± 44 Nm while isokinetic peak torque was 94 ± 37 Nm, with significant correlations observed between the two strength measurements and WOMAC function (r = 0.618, r = 0.663) as well as walking speed (r = 0.641–0.710) [23,24]. Similarly, in this study, a strong correlation was found between the isometric peak torque measured using the IB-LS and the isokinetic peak torque obtained from the CSMI device. This finding suggests that both strength assessment methods can serve as important indicators in evaluating physical function.

Muscle strength assessment using the IB-LS device is a fast, convenient, and highly reliable evaluation method, making it suitable for clinical applications. Prior to each session, the IB-LS was calibrated using a standard load-based procedure provided by the manufacturer. This process was essential to reduce measurement bias and ensure consistency, particularly considering the structural differences from the CSMI system. Compared to the CSMI device, the IB-LS offers advantages such as simpler initial calibration, fewer spatial constraints, and greater portability. These features support its applicability in both clinical and community-based settings. The IB-LS device required less than five minutes for setup and testing per participant. No usability issues were reported, and all participants were able to complete the testing with minimal guidance. This suggests that the IB-LS device is feasible for older adults and suitable for use in clinical and community settings [25]. To ensure measurement accuracy and consistency, the IB-LS was calibrated prior to each testing session using a standardized load-based protocol recommended by the manufacturer, which helped to minimize systematic error and ensure comparability with the CSMI system. Additionally, knee isometric strength has shown significant correlations with walking speed and the Timed Up and Go test, supporting its role as an essential tool for lower limb function assessment [26].

However, this study has several limitations. First, the relatively small sample size limits the generalizability of the findings, and future studies should include a more diverse population considering different age groups and physical conditions. Second, the influence of participants’ exercise experience on strength measurement results was not controlled, necessitating further research on this factor. Third, follow-up studies are needed to determine whether the IB-LS device can be utilized for the long-term monitoring of muscle strength changes. Fourth, this study assessed isokinetic strength at a single velocity (60°/s), and additional analyses at different speeds may be required. Fifth, further research is needed to evaluate the practical applicability of the IB-LS device in various clinical settings. Sixth, all participants in this study were recruited from a single geographic area (D Metropolitan City, Republic of Korea), which may limit the generalizability of the results to broader or more diverse populations. Future studies should consider regionally or ethnically diverse samples to validate the findings across different demographic groups.

## 5. Conclusions

This study confirmed that the IB-LS device demonstrates high test–retest reliability and validity in assessing knee isometric strength in older adults and shows moderate to strong agreement with conventional isokinetic strength assessment using the CSMI device. While the results support the potential of the IB-LS as a feasible tool for strength evaluation in both clinical and research settings, the relatively wide limits of agreement observed in certain comparisons—particularly in right knee extension—warrant cautious interpretation in clinical applications. Given the modest sample size and limited participant diversity, further studies involving larger and more heterogeneous populations are necessary to strengthen the generalizability of the findings and to explore the device’s long-term usability. Additionally, due to its operational simplicity and portability, the IB-LS may offer advantages for use in community-based settings or preliminary screenings for sarcopenia in older adults. However, its broader role in assessing overall physical function should be investigated further through targeted validation studies.

## Figures and Tables

**Table 1:** Participant’s characteristics (*n* = 21).

	M ± SD ^1^	*t* (*p*)
Sex (Male/Female)	11/10	0.213 (0.833)
Age (Years)	65.10 ± 4.56	65.422 (<0.001)
Height (cm)	161.62 ± 7.59	97.525 (<0.001)
Weight (kg)	65.91 ± 10.92	27.647 (<0.001)
Dominant Side (Left/Right)	0/21	
Left Leg Length (cm)	36.38 ± 2.72	61.193 (<0.001)
Right Leg Length (cm)	36.31 ± 2.64	62.949 (<0.001)
BMI	25.24 ± 3.46	34.241 (<0.001)
Muscle Mass (kg)	25.95 ± 6.36	19.142 (<0.001)
Left Grip Strength (kg)	32.86 ± 11.42	13.069 (<0.001)
Right Grip Strength (kg)	33.62 ± 10.21	15.149 (<0.001)

^1^ Mean ± Standard Deviation.

**Table 2:** Intraclass Correlation Coefficients of knee joint isometric muscle peak force (*n* = 21).

		IB-LS Peak Force (kg)			ICC
		1st Trial	2nd Trial	3rd Trial	
Left	Flexion	18.92 ± 6.79	18.16 ± 7.38	18.25 ± 6.95	0.982
	Extension	30.02 ± 9.54	30.50 ± 13.07	31.68 ± 12.63	0.975
Right	Flexion	20.36 ± 7.65	19.62 ± 7.69	19.51 ± 7.25	0.987
	Extension	29.57 ± 10.85	33.15 ± 11.00	32.97 ± 11.11	0.856

**Table 3:** Validity of knee flexion and extension isometric peak torque of IB-LS and CSMI devices (*n* = 21).

		IB-LS Isometric Peak Torque (Nm)	CSMI Isometric Peak Torque (Nm)	*t* (*p*)	ICC
Left	Flexion	76.04 ± 31.97	64.00 ± 24.86	4.244 (<0.001)	0.946
	Extension	118.60 ± 46.00	101.48 ± 42.18	2.310 (0.032)	0.826
Right	Flexion	71.05 ± 28.10	63.67 ± 24.16	2.846 (0.010)	0.946
	Extension	125.08 ± 45.81	106.48 ± 41.49	3.010 (0.007)	0.883

**Table 4:** Validity of knee flexion and extension isometric peak torque of IB-LS and isokinetic 60°/s of CSMI devices (*n* = 17).

		IB-LS Isometric Peak Torque (Nm)	CSMI Isokinetic 60°/s Peak Torque (Nm)	*t* (*p*)	ICC
Left	Flexion	69.78 ± 30.63	53.39 ± 25.92	3.655 (0.002)	0.881
	Extension	108.09 ± 40.07	86.32 ± 38.37	3.140 (0.006)	0.847
Right	Flexion	65.98 ± 28.29	44.43 ± 23.39	4.254 (0.001)	0.807
	Extension	117.98 ± 45.35	74.08 ± 37.76	5.062 (<0.001)	0.775

**Table 5:** Agreement of knee flexion and extension peak torque between IB-LS and CSMI devices: Bland–Altman analysis.

			MD (Nm) ^1^	95% CI	LoA	*p*
Isometric Peak Torque (Nm)	Left	Flexion	13.2	−25.49 to 56.45	−55.8 to 82.2	<0.001
		Extension	74.7	−6.18 to 155.66	−6.2 to 155.6	<0.001
	Right	Flexion	28.1	−34.52 to 75.17	−34.5 to 90.8	<0.001
		Extension	93.5	−30.27 to 167.78	−5.6 to 192.5	<0.001
Isokinetic Peak Torque (Nm)	Left	Flexion	38.2	29.11 to 47.38	−16.24 to 92.7	<0.001
		Extension	48.4	38.28 to 58.43	−11.71 to 108.4	<0.001
	Right	Flexion	31.0	21.07 to 41.03	−28.46 to 90.6	<0.001
		Extension	56.0	42.09 to 69.91	−26.91 to 138.9	<0.001

^1^ Mean difference.

## Data Availability

The data supporting the reported results are not publicly available due to privacy and ethical restrictions.
